# The use of polyphenols extracted from Chinese sweet leaf tea (*Rubus suavissimus* S. Lee.) as novel drugs for the treatment of metabolic dysfunction associated steatotic liver disease

**DOI:** 10.1186/s41065-025-00504-6

**Published:** 2025-07-24

**Authors:** Yu Pan, Yu Liu, Yixuan Huo, Suren R Sooranna, Lu Chen, Lijun Yin, Zhigang Yan, Danna Huang, Lihe Jiang, Wuwei Wu

**Affiliations:** 1 National Engineering Research Center for Southwest Endangered Medicinal Resource Development, Guangxi Botanical Garden of Medicinal Plants, Nanning, 530023 China; 2 School of Basic Medical Sciences, Youjiang Medical University for Nationalities, Baise, Guangxi 533000 China; 3 Academic Department of Obstetrics and Gvnaecology, Chelsea and Westminster Hospital, Imperial College London, 369 Fulham Road, London, SW10 9NH UK; 4 Hebei Key Laboratory of Nutrition and Health, Shijiazhuang, Hebei 050000 China; 5 Wuming Hospital, Guangxi Medical University, Nanning, 530100 China

**Keywords:** Chinese sweet leaf tea, Polyphenols, Medical small molecules, Multifaceted approach, Metabolic dysfunction associated steatotic liver disease, Hypolipidemic effects

## Abstract

**Background:**

Metabolic dysfunction associated steatotic liver disease (MASLD) is a pressing global health issue with limited treatment options. Chinese sweet leaf tea (CSLT), rich in polyphenols, shows promise in addressing MASLD due to its anti-inflammatory, obesity-reducing, and metabolic-regulating properties. This study aimed to explore the therapeutic potential of CSLT polyphenols for MASLD treatment.

**Methods:**

A comprehensive approach combining bioinformatics, network pharmacology, molecular docking, high-throughput sequencing, and experimental validation was employed. CSLT extracts were screened for active compounds, and potential targets associated with MASLD were predicted. A protein-protein interaction network was constructed to identify key regulators of lipid metabolism. Molecular docking studies validated interactions between core polyphenols and MASLD-related targets. In vitro studies using HepG2 cells and in vivo studies in a high-fat diet-induced rat model were conducted to assess the efficacy of polyphenol-rich CSLT extract (PE-CSLT) in ameliorating MASLD symptoms.

**Results:**

A total of Eighty-two chemical constituents were initially identified in CSLT. Twenty-six active compounds were screened in CSLT extracts, targeting one hundred and six proteins related to twenty MASLD-associated genes. PE-CSLT demonstrated significant efficacy in reducing body weight gain, serum lipid levels, and liver fat accumulation in MASLD rats. Gene expression analysis revealed modulation of key metabolic regulators, including SREBP1, ACACA, AMPK, and PPARα. Molecular docking confirmed strong binding of eleven PE-CSLT components to these targets. Our findings highlight the therapeutic potential of PE-CSLT for MASLD, providing a scientific basis for its development as a novel drug.

**Conclusion:**

This study underscores the promise of natural products in modern medicinal practices and emphasizes the need for further clinical evaluation of PE-CSLT in MASLD management.

**Graphical abstract:**

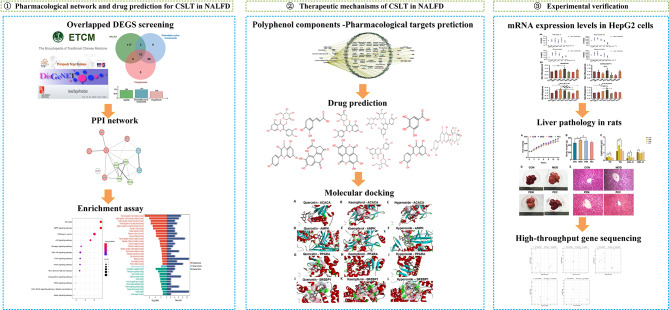

**Supplementary Information:**

The online version contains supplementary material available at 10.1186/s41065-025-00504-6.

## Introduction

Nonalcoholic fatty liver disease (NAFLD) was historically defined by the exclusion of secondary causes and significant alcohol use, while metabolic dysfunction-associated steatotic liver disease (MASLD), its 2023 reclassification, emphasizes metabolic risk factors (e.g., obesity, diabetes) as core diagnostic criteria [1]. Both share pathological features, including hepatic steatosis, insulin resistance, and cardiometabolic risks, and advocate lifestyle interventions as primary management [2]. MASLD refines patient stratification by requiring metabolic comorbidities, improving prognostic relevance for fibrosis and mortality, yet retains NAFLD’s foundational pathological mechanisms [3]. Despite its significant clinical burden, the therapeutic options for MASLD remain limited, underscoring the imperative for innovative strategies targeting metabolic dysfunction, inflammation, and fibrotic progression to address this evolving global health challenge [4]. Traditional Chinese Medicine (TCM), which is relatively inexpensive and readily available, have been shown to be effective in the prevention and treatment of chronic liver disease in China and others countries.

Recent research has focused on tapping into the potential of natural products, particularly polyphenols, for the management of MASLD. Among these, Chinese sweet leaf tea (CSLT; *Rubus suavissimus* S. Lee.), a traditional herbal beverage, has garnered attention due to its rich polyphenol content and reported health benefits [5]. Recent studies have shown that CSLT has a significant effect on its ability to lower blood lipids and cholesterol levels and it can help to maintain a balanced lipid metabolism [6]. The major active components of CSLT have been shown to be polyphenols (8.94%), rubusoside (4%~5%) and flavonoids (≥ 3.2%) [7]. However, previous studies on CSLT have primarily focused on its anti-inflammatory, anti-obesity, and glucose-regulating effects, with limited exploration into its therapeutic potential for MASLD [8, 9].

While the existing literature on CSLT demonstrates promising results in mitigating low-grade inflammation, improving obesity phenotypes, and restoring metabolic homeostasis, several limitations persist. Firstly, the majority of studies have utilized animal models, with limited translation to human clinical settings. Secondly, the specific mechanisms underlying the therapeutic effects of polyphenols extracted from CSLT (PE-CSLT) in MASLD remain elusive. Lastly, the studies have not comprehensively evaluated the polyphenol composition and their individual contributions to the observed pharmacological effects. Specifically, the biochemical processes through which CSLT-derived polyphenols influence fat metabolism remain unknown.

**Fig. 1:**
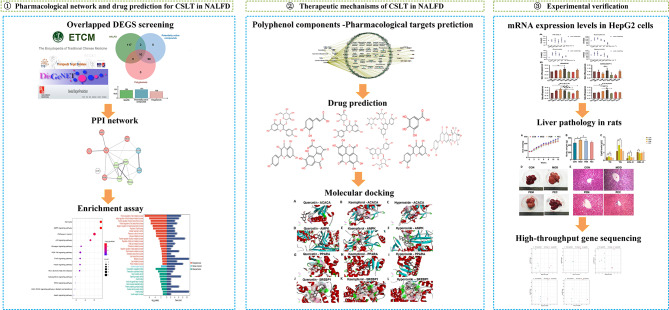
Workflow of the study

To bridge these gaps, our study employs a multifaceted approach that integrates bioinformatics, network pharmacology, molecular docking, high-throughput sequencing technology, and experimental validation (Fig. 1). This comprehensive methodology aims to thoroughly examine the therapeutic potential of PE-CSLT in MASLD. Our innovative strategy facilitates the identification of novel targets and signaling pathways implicated in MASLD treatment, thereby enhancing our understanding of the underlying mechanisms. This comprehensive approach not only uncovers new targets and signaling pathways but also offers a profound understanding of the molecular mechanisms that drive the therapeutic effects of PE-CSLT. Our work lays the groundwork for the development of novel therapeutic approaches for MASLD and underscores the promise of natural products in modern medicinal practices.

## Materials and methods

### Construction of ingredients database

The information regarding the small compounds (MW ≤ 900 Da) in CSLT were obtained from China National Knowledge Infrastructure (CNKI) (https://en.wikipedia.org/wiki/CNKI; https://www.cnki.net/) and PubMed databases (https://pubmed.ncbi.nlm.nih.gov/). The information was then filtered with respect to their absorption, distribution, metabolism, excretion and toxicity (ADMET) properties based on their oral bio-availability (OB) ≥ 30% and drug likeness (DL) ≥ 0.18. Finally, eighty-two chemical CLST-derived compounds were selected from the PubChem (https://pubchem.ncbi.nlm.nih.gov/) and the Encyclopedia of Traditional Chinese Medicine (ETCM; http://www.tcmip.cn/ETCM/index.php/Home/) databases.

### Potential targets prediction in relation to CSLT

The potential protein targets of the eighty-two chemical molecules from CSLT were found in the Swiss Target Prediction database (http://www.swisstargetprediction.ch/index.php) based on a combination of their 2D and 3D similarities (probability ≥ 0.5). Then, one hundred and twenty-nine MASLD-related targets were obtained from the Disgenet database (https://www.disgenet.org/) with GDA score ≥ 0.30 and the TTD database (http://db.idrblab.net/ttd/) using the keywords “Non-alcoholic Fatty Liver Disease” (C0400966), “Fatty Liver Disease, Nonalcoholic, Susceptibility To, 1” (C2750440), “Fatty Liver Disease, Nonalcoholic, Susceptibility To, 2” (C3150651). Twelve MASLD-related targets were selected.

### Analysis of Protein-Protein interaction (PPI) cluster network

The obtained target proteins were imported into the STRING database (https://string-db.org/) to construct a PPI network. A required score of 0.40 and an FDR stringency of ≤ 0.05 were employed to ensure high confidence in the interactions. Subsequently, the Markov Cluster Algorithm (MCL) was utilized for further analysis of the PPI network, with the MCL inflation parameter set to 3, determining the granularity of the clusters within the network. This clustering analysis facilitated the identification of protein groups with similar functions and roles in the context of CSLT treatment for MASLD.

### Gene ontology enrichment analysis of the MASLD-associated targets

The biological function of MASLD-targets related with CSLT were analyzed by using the DAVID 6.8 (https://david.ncifcrf.gov/) and PANTHER databases (http://pantherdb.org/). The target related pathways were acquired from the KEGG database (http://www.genome.jp/kegg/).

### Construction of a CSLT-MASLD “ingredients-targets-pathways” interaction network

The interaction data for the polyphenolic compounds and targets in CSLT against MASLD were visualized by using Cytoscape software, version 3.10.2 (Boston, MA, USA). Three important topological features including “Degress”, “Betweenness centrality” and “Closeness centrality”, were selected within the network analyzer plugins in Cytoscape software.

### Molecular docking and screening [10]

Eleven core polyphenolic compounds with a Druglikeness Weight of at least 0.5 were selected from the aforementioned network diagram. The 3D structures of these compounds were retrieved from PubChem (https://pubchem.ncbi.nlm.nih.gov/) and converted into the required format using OpenBabel 2.4.1 software. For the four core target proteins identified in the network diagram, their 3D crystal structures were obtained from the RCSB PDB database (https://www.rcsb.org/), with selection criteria including Homo sapiens, X-ray diffraction, and a resolution (Å) of ≤ 2.5. Small molecular ligands that had co-crystallized structures with the target proteins were identified. Using Autodocking Tools 1.5.6 software, all small molecular ligand structures underwent energy minimization, force field application, and conformational optimization. Receptor structures were prepared by adding hydrogens, assigning charges, removing water molecules, and modifying amino acid residues. Potential active pockets and binding sites of the target proteins were determined, and molecular docking calculations were performed to screen for small molecule ligand-protein complexes with good affinities. Finally, Discovery Studio Client 4.5 software was used to visualize the optimal conformations of the ligand-target protein complexes.

### Chemicals and reagents

Simvastatin (SIM) was purchased from Hangzhou MSD Pharmaceutical Co., Ltd. (Hangzhou, China). Dullbecco’s modified Eagle’s medium (DMEM) and fetal bovine serum (FBS) were purchased from Thermo-Fisher Biochemical Products (Beijing) Co., Ltd. (China). Total cholesterol (TC), triglyceride (TG), HDL-cholesterol (HDL-C), HDL-cholesterol (LDL-C), alanine aminotransferase (ALT) and aspartic transaminase (AST) was assayed using kits purchased from Nanjing Jiancheng Bioengineering Institute (Nanjing, China). Thiazolyl blue tetrazolium bromide (MTT) was purchased from Beijing Solarbio Science & Technology Co.,Ltd. (Beijing, China).

### Preparation of PE-CSLT

Dried CSLT leaves were collected from Dayao Mountain, Jinxiu County, Guangxi, China. A voucher specimen of this material was deposited in the National Engineering Research Center for Southwest Endangered Medicinal Resource Development (ID: IBK00286029). It was identified as a dry product of Chinese sweet leaf tea by Professor Mingsheng Lan form Guangxi Botanical Garden of Medicinal Plants The crushed CSLT leaves were immersed with distilled water at a solid liquid ratio of 1:10 (m/v), which was then heated and refluxed twice, each for 2 h. The extracts were then filtered, and the filtrates were combined and concentrated under reduced pressure. The concentrated solution was loaded onto a column with D101 macroporous resin to separate the PE-CSLT. The column was washed with water and 20% ethanol successively, and after that the PE-CSLT was eluted with 50% ethanol and it was concentrated under reduced pressure. The obtained PE-CSLT was assayed using Folin’s reagent to determine the concentration of polyphenols which was 54.62%.

### Establishment of the rat MASLD model

Adult male Sprague-Dawley (SD) rats (210 ~ 230 g) were randomly divided into four groups: the control group (CON), the high-fat diet model group (MOD), the PE-CSLT control group (PEC) and the PE-CSLT model group (PEM). The control group and the PE-CSLT control group were fed with basic formula diet for normal growth, and the model group and the PE-CSLT model group were induced with a high fat diet (HFD: 78.8% ordinary feed, 10% lard, 10% egg yolk powder, 1% cholesterol and 0.2% cholate) to establish the MASLD model.

To optimize resource allocation within the six-month project timeline, a preliminary dose-range pilot study was performed using a MASLD rat model. Animals were administered CSLT polyphenols at three doses: low (1.8 g/kg·d), medium (3.6 g/kg·d), and high (7.2 g/kg·d). Histological analysis revealed no detectable hepatic structural improvements at the low and medium doses, with corresponding minimal changes in metabolic biomarkers. Based on these preliminary findings, subsequent formal in vivo efficacy evaluations were limited to the high-dose regimen (7.2 g/kg·d).

The PE-CSLT control group and the PE-CSLT model group received with PE-CSLT (a dose equivalent to the volume of 7.2 g/kg crude drug per day) by gavage, and the control group and the model group received an equivalent amount of saline (10 ml/kg). After 12 weeks of continuous treatment, the rats were fasted for 12 h, with water still available, following the final administration. The rats were then anesthetized with 2% pentobarbital sodium (50 mg/kg). Blood was subsequently collected from their abdominal aortas, centrifuged at 4 °C at 1000 g for 20 min, and the serum was obtained and stored at -20 °C. The livers of the rats were also removed for further analysis. All experimental procedures were approved and conducted in accordance with the National Institutes of Health Guide for the Care and Use of Laboratory Animal and the guidelines of the Animal Experimentation Ethics Committee of Guangxi Botanical Garden of Medicinal Plants.

### Cell culture and treatments

The HepG2 human cell line (ATCC HB-8065™) was obtained from Experimental Animal Center of Sun Yat-Sen University (Guangzhou, China). The cells were incubated overnight in DMEM with 0.1% FBS and 1% antibiotics (penicillin-streptomycin) at 37 °C in a 5% humidified CO_2_ incubator. The medium was routinely changed every 3 days, and the cells were passaged by trypsinization when there were 80% confluent. A fatty HepG2 cell model was induced by addition of a sodium oleate solution according to the method reported previously with slight modifications [11, 12]. Briefly, HepG2 cells were treated with 0.2 mM sodium oleate solution to induce excessive fat synthesis. After the fatty HepG2 cell model was established by assessing the levels of TC and TG, they were divided into 7 groups and treated as follows: the control group (CON) which had 10% FBS, the model group (MOD) which of fatty HepG2 cells, the simvastatin group (SIM), where the fatty HepG2 cells were treated with the positive drug, simvastatin, and control HepG2 cells that were treated with PE-CSLT (PET), kaempferol (KAE), quercetin (QUE) and hyperoside (HYP), respectively.

### Measurement of cell inhibition rate

The cytotoxicity of PEC at a range of doses (6.69, 13.38, 26.75, 53.50 and 107.00 µg/mL) was measured by using the MTT (5 mg/mL) colorimetric assay. The cells were incubated with rat sera containing PEC after 12 h. The fatty HepG2 cells were washed with pH7.0 phosphate-buffered saline (PBS) and treated with 150 µL of dimethyl sulphoxide (DMSO) for 4 h. Then, the TC and TG contents of the cells were determined by using commercial kits. The analyses were performed in six replicate wells, and each experiment was repeated 3 times. The cell inhibition ratio (%) (IR) was determined as follows: Inhibition Ratio (%) = [(OD control − OD treated)/(OD control − OD blank)] ×100%.

### Gene expression analysis

Real-time-qPCR: Total RNA was isolated from HepG2 cells by using TRIzol (Transgen, Beijing, China)/RNeasy Lipid Tissue Kit (Qiagen), and these were converted to cDNA using oligo (dT). cDNA templates were amplified using real-time PCR with the iTaq SYBR Green Supermix (Bio-Rad, Hercules CA). The rat primer sequences used are showed in Supplementary Table S1.

High-throughput gene sequencing (Illumina-HTGS): Total RNA was isolated from rat livers and this was processed and sequenced by Novogene Bioinformatics Technology Co. Ltd. (Beijing, China). The RNA-seq results were analyzed to determine the differentially expressed genes among the four experimental groups—CON, MOD, PEC and PEM groups. The different genes related to lipid metabolism were screened according to their|log_2_Fold change| ≥1, FDR < 0.001.

### Data analysis

GraphPad Prism 8.0 and SPSS 21 software packages were used for statistical analysis. Continuous variables were presented as the means ± standard deviations (SDs). Statistical differences between groups were evaluated by one-way analysis of variance (ANOVA) followed by the Tukey’s mulitple compasions test. Differences were considered statistically significant when *p* < 0.05.

## Results

### Screening of MASLD-related targets

**Fig. 2:**
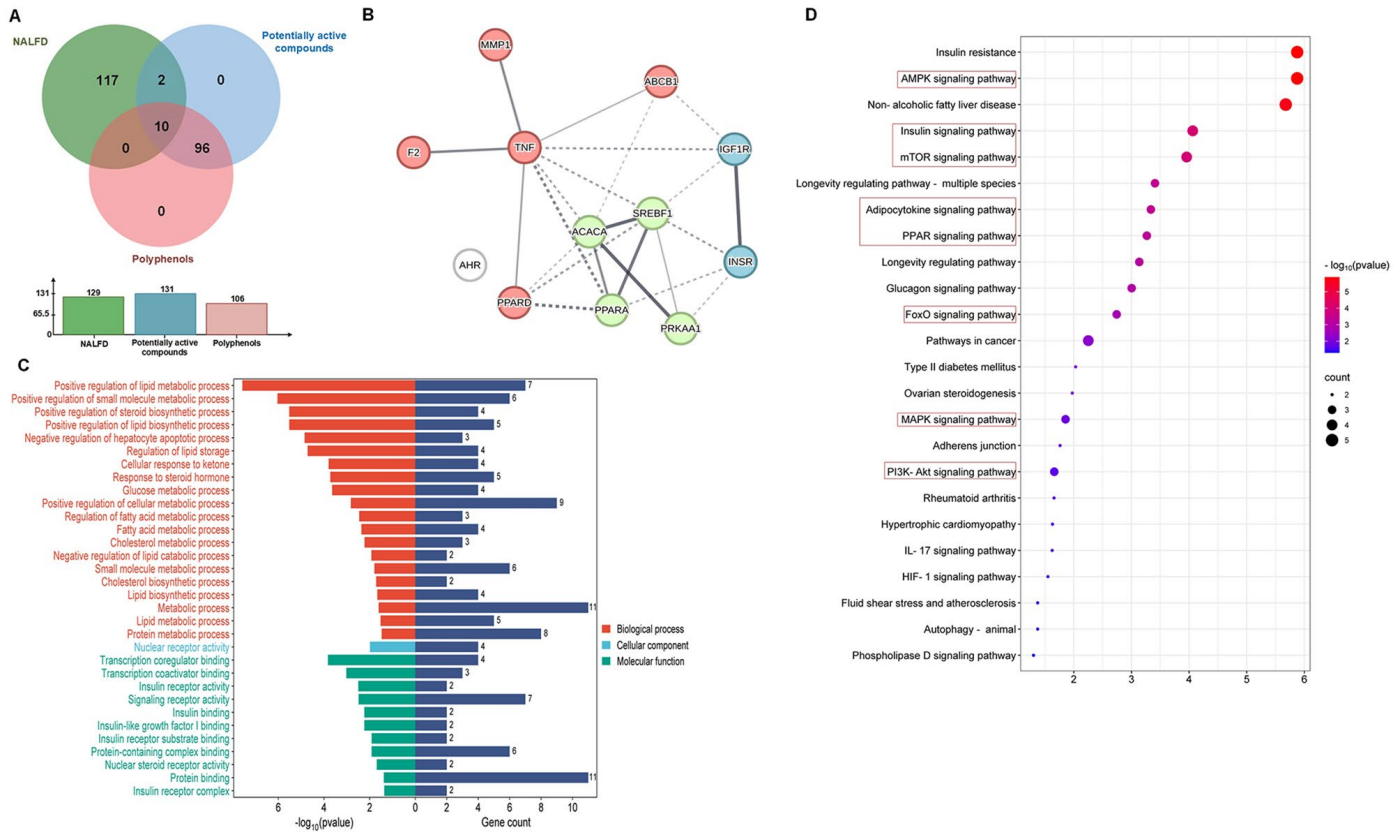
The biological functional enrichment analysis of CSLT related to MASLD. (**A**) Target screening for CSLT in the treatment of MASLD. (**B**) PPI network analysis of twelve targets for CSLT in the treatment of NALFD. (**C**) Analysis of biological processes and functions for CSLT in the treatment of MASLD. (**D**) Signaling pathway analysis of CSLT in the treatment of MASLD

Eighty-two chemical compounds in CSLT were screened to pinpoint the bioactive ingredients. Among them, twenty-six compounds exhibited favorable properties concerning ADMET absorption levels, drug-likeness weights, and bioavailability scores. These compounds mostly belonged to the polyphenol category, featuring one or multiple phenolic structures. By eliminating duplicate targets and employing molecular similarity matching algorithms, one hundred and thirty-one potential targets were identified. Upon comparing these potential targets from CSLT with the one hundred and twenty-nine MASLD-related candidate targets, twelve putative targets were discovered to be common between them (Fig. 2A).

### MASLD-related biological functional enrichment analysis

The PPI network uncovered multiple clusters focused on critical metabolic regulators, namely INSR, PRKAA1/AMPK, SREBAF1/SREBP1, and PPARα. These proteins are recognized for their essential roles in lipid metabolism, insulin signaling, and maintaining cellular energy homeostasis. Within this MASLD-associated network, proteins with higher node degrees, such as TNF (degree: 8), SREBF1 (degree: 7), ACACA (degree: 6), and PPARα (degree: 5), stood out as key players (Fig. 2B). These hubs are potentially crucial modulators of the disease phenotype due to their interactions with numerous other proteins. Conversely, AHR showed no observed interactions (degree: 0).

Remarkably, the terms ‘Positive regulation of lipid metabolic process’ (GO:0045834) and ‘Positive regulation of small molecule metabolic process’ (GO:0062013) were notably enriched. The observed gene counts surpassed background expectations by over 1.8-fold (Fig. 2C and D), implying that dysregulation of these processes significantly contributes to MASLD progression.

Multiple pathways and GO terms exhibited low False Discovery Rates (FDRs), highlighting their strong connection to MASLD. For instance, the AMPK signaling pathway (FDR = 1.33E-06) and ‘Positive regulation of lipid metabolic process’ (FDR = 2.67E-08) were particularly relevant. Among these, genes such as TNF, SREBF1, AMPK, PPARD, and IGF1R consistently emerged as significant matching proteins within our network, further emphasizing their functional significance in MASLD. Proteins linked to lipid metabolism and biosynthesis, including SREBF1, PPARα, ACACA, and PPARD, were enriched in processes tied to lipid accumulation, a defining feature of MASLD.

### Complex biological network analysis of CSLT

Among the analyzed polyphenol components, quercetin stands out as the top-ranked compound, boasting the highest Closeness Centrality (0.55686) and Degree (72). This indicates its significant role in the biological network, reflecting its strong potential for interacting with numerous targets and pathways, thus marking it as a prominent therapeutic candidate for MASLD. Other notable polyphenol components within the top six include kaempferol, hyperoside, isoquercitrin, brevifolic acid, and ellagic acid (Table 1). These polyphenolic compounds exhibit elevated Closeness Centrality and Degree values, suggesting their substantial contributions to the network’s connectivity and potential therapeutic importance in MASLD (Supplementary Table S2).

**Table 1:**
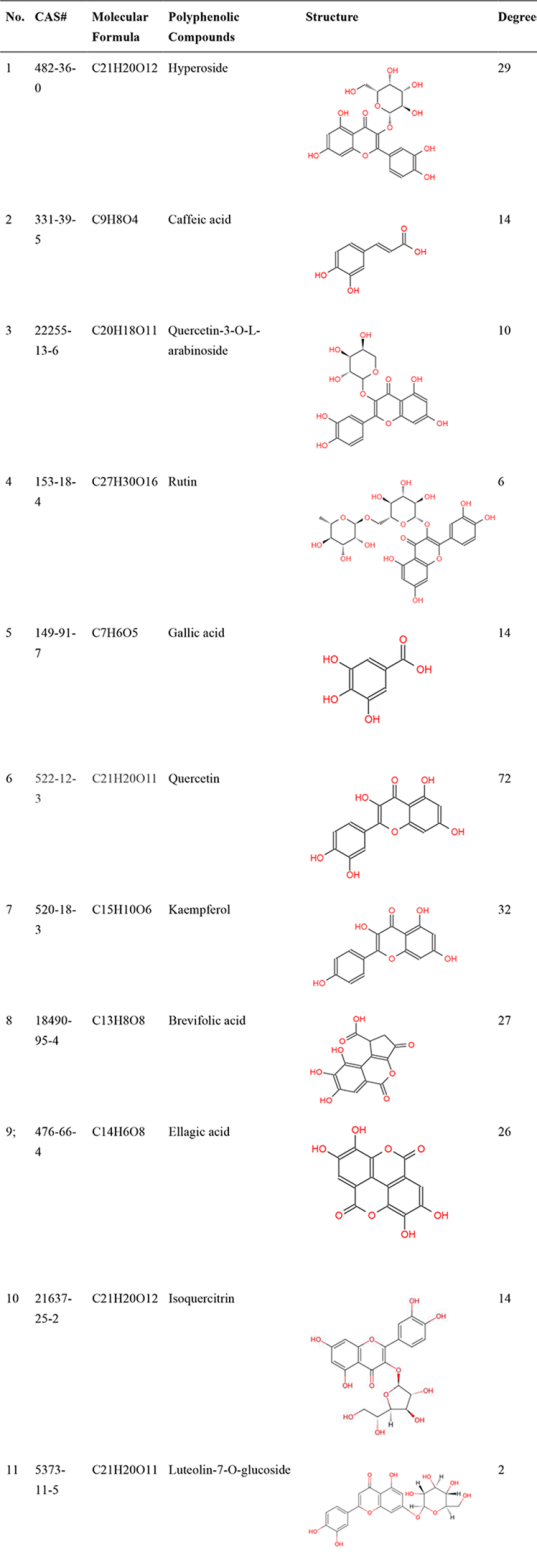
The polyphenols compounds isolated from Chinese sweet leaf tea

**Table 2:** The binding energies of the core active polyphenols compouds with their corresponding core targets (kcal/mol)

Compound	PubChem CID	AMPK/PRKAA2	SREBAF1/SREBP1	ACACA	PPARA
		7MYJ	1AM9	4Y2G	6KAX
Hyperoside	5,281,643	-5.9	-6.6	-5.8	-6.6
Quercetin	5,280,343	-4.9	-5.3	-4.9	-5.2
Kaempferol	5,280,863	-4.7	-5.0	-4.7	-5.0

**Fig. 3:**
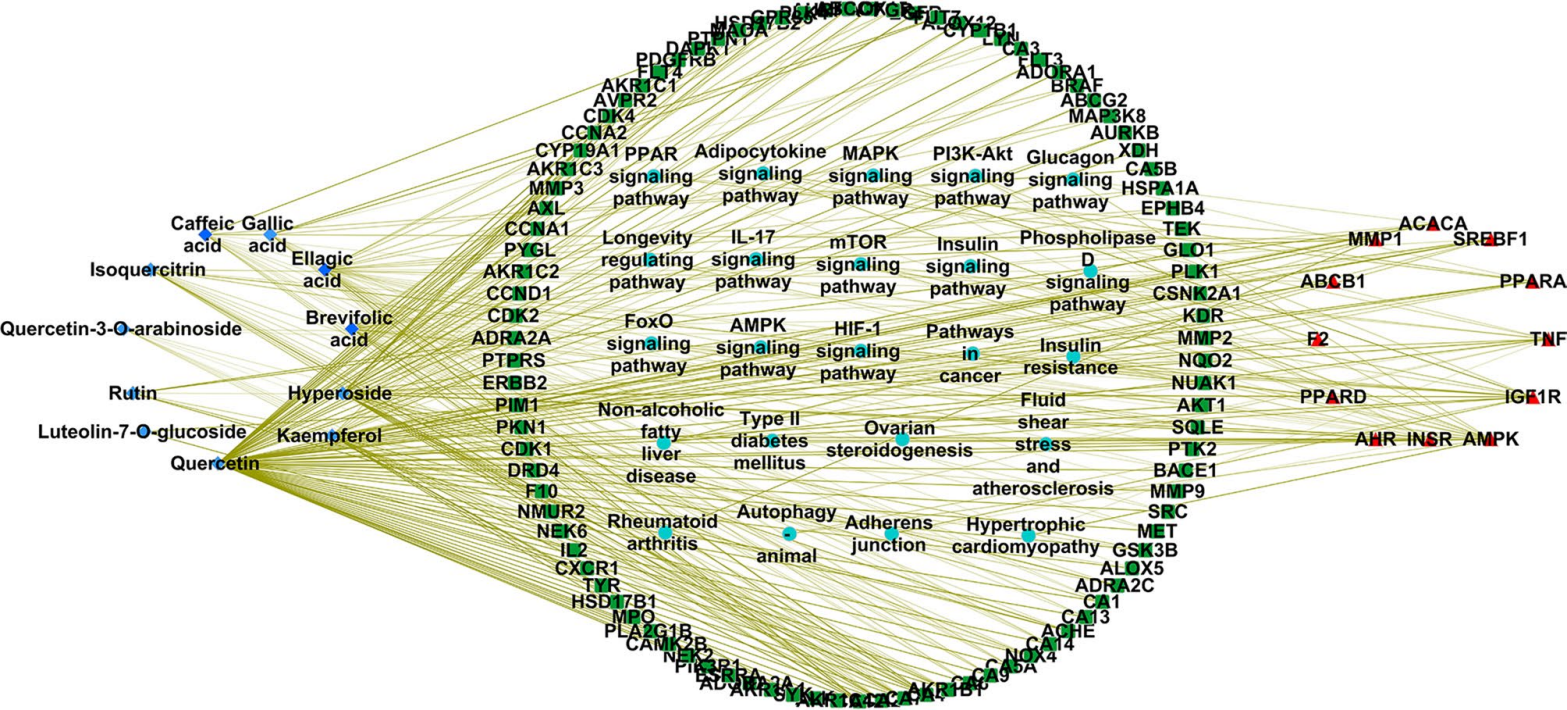
A “polyphenol compounds-potential targets-signal pathway” network. The blue, red, cyan nodes represent the polyphenols of CSLT, the potential targets related to the polyphenols, the MASLD targets related the polyphenols and the signaling pathways associated with the MASLD targets, respectively

At the gene level, INSR and IGF1R emerge as key players with high Degrees (15 and 13, respectively) and Closeness Centralities (0.3198 and 0.4201). This underscores their critical roles in mediating insulin signaling and growth factor responses. SREBF1, a master lipid homeostasis regulator, also holds a prominent position due to its centrality and high connectivity. Other prominent genes include PRKAA1, TNF, and PPARα all implicated in MASLD pathogenesis and exerting significant network influence (Fig. 3).

The AMPK signaling pathway, renowned for its role in energy metabolism and lipid synthesis regulation, holds a central position, involving multiple key nodes. Both the Insulin signaling and PI3K-Akt signaling pathways, essential for glucose homeostasis and cell growth, also feature prominently. These pathways closely interact with key small molecules and genes, reinforcing their therapeutic potential in MASLD. Other notable pathways include mTOR signaling, regulating cellular growth and proliferation, and the MASLD pathway itself, directly addressing the disease process (Fig. 3).

### Molecular docking of the PE-CSLT core components and the MASLD targets

**Fig. 4:**
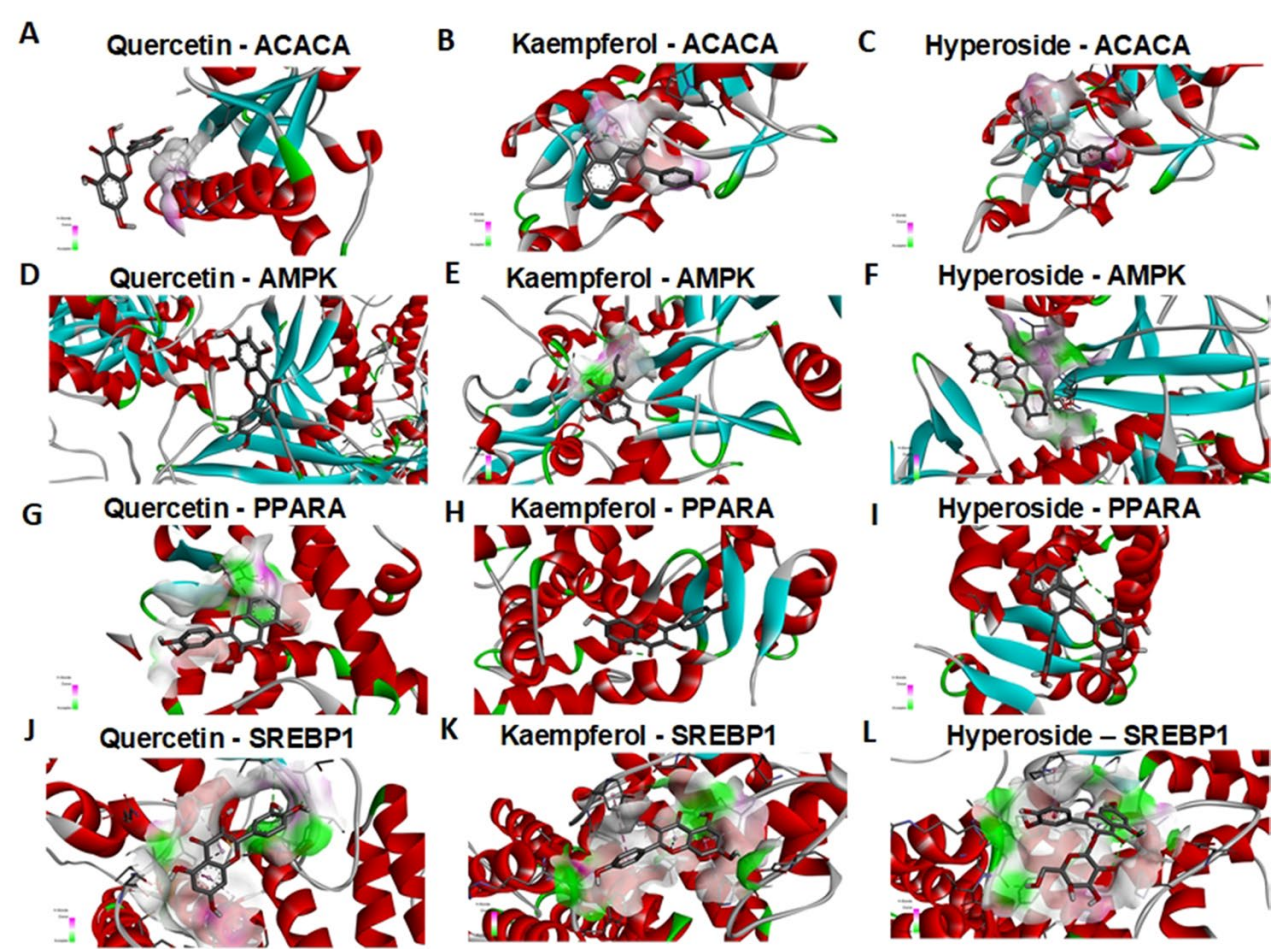
The molecular interaction.(**A-L**) The three-dimensional molecular docking of the three core polyphenols compounds of CSLT in the treatment of MASLD with their respective four core target proteins. The filtering criteria used were a Druglikeness Score ≥ 0.5 and a binding energy ≤ -5.0 kcal mol

To validate the activities of the eleven polyphenol components identified from the complex biological network of CSLT and confirm their docking conformations with the twelve core target proteins, as indicated by the network pharmacology analysis, we performed protein affinity tests. These tests focused on the three most critical polyphenol compounds with a Druglikeness Score of 0.5 or higher (Table 2). It is generally accepted that a binding energy of -5.0 kcal/mol or lower indicates significant binding activity between a small ligand molecule and its protein receptor [13]. The results showed that the three ligand molecules had favorable binding affinities with the four protein receptors, AMPK, ACACA, SREBP1, and PPARα (Fig. 4A-L). Hyperoside, Quercetin, and Kaempferol exhibited binding energies of -5.0 kcal/mol or lower with SREBP1 protein receptors. Hyperoside, Quercetin, and Kaempferol demonstrated binding energies of -7.0 kcal/mol or lower with PPARα protein targets, indicating strong binding activities. Specifically, Quercetin and Kaempferol exhibited a binding energy of -8.1 kcal/mol and − 8.0 kcal/mol with AMPK, respectively.

### Verification of the effects of PEC-CSLT on HepG2 cell viability

**Fig. 5:**
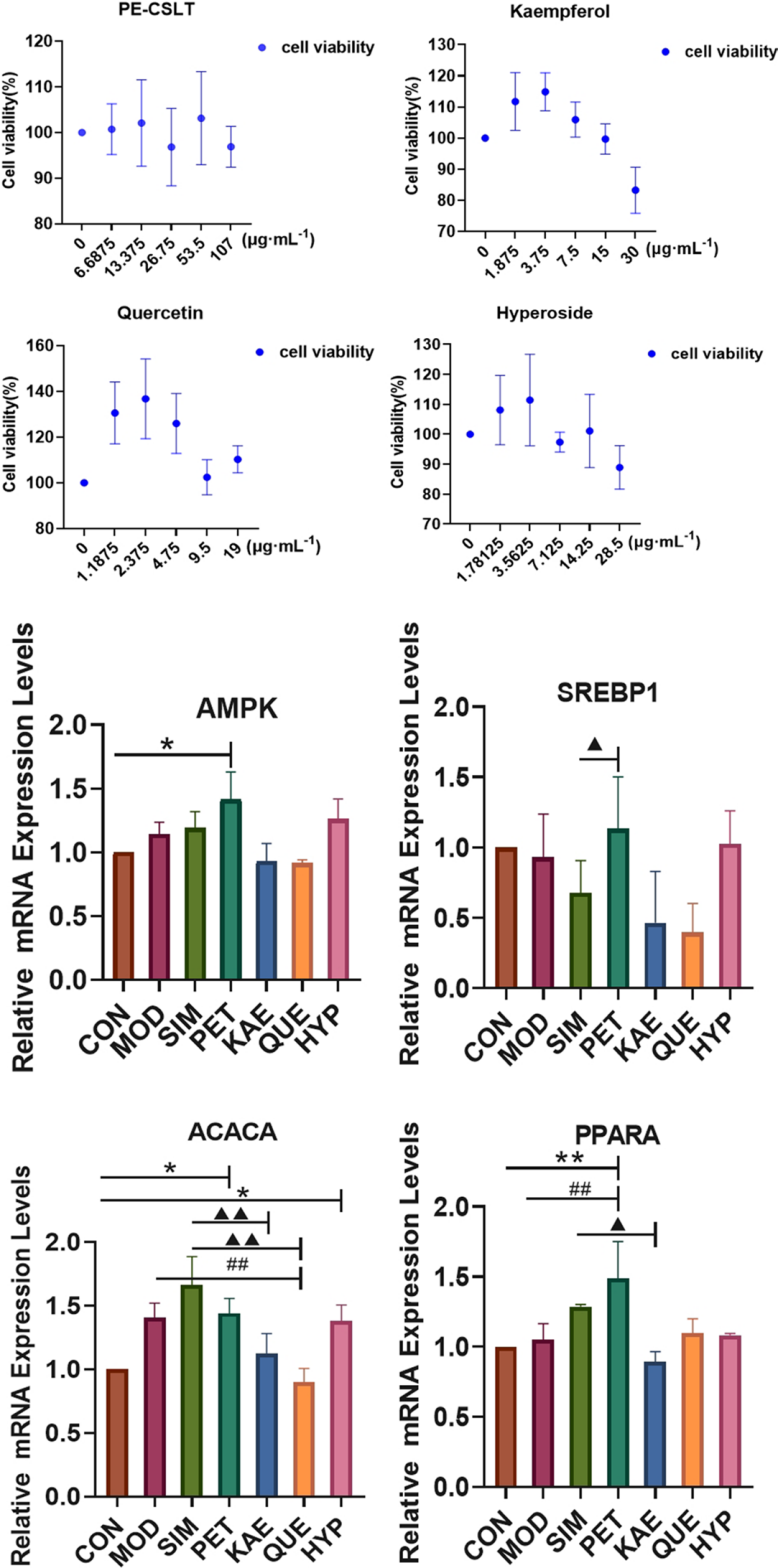
The effects of extracts from CSLT on lipid metabolism in NALFD in steatotic HepG2 cells. (**A**) The MTT assay was used to screen the optimal concentration of polyphenols extracted from CSLT that would cause observable changes in HepG2 cells. (**B**) The effects of polyphenols extracted from CSLT on the mRNA expression of genes related to lipid metabolism in HepG2 cells. The data are expressed as means ± SDs (*n* = 3). *p < 0.05 and **p < 0.01 when compared with the CON group; ^**##**^*p* < 0.01 when compared with the MOD group; ^▲^*p* < 0.05, ^▲▲^*p* < 0.01 and ^▲▲▲^*p* < 0.001 when compared with SIM group. CON: control, MOD: model, SIM: simvastatin, PEC: PE-CSLT, KAE: Kaempferol, QUE: Quercetin, HYP: Hyperoside

To validate that three polyphenol compounds (KAE, QUE and HYE) were associated with AMPK, SREBP1, ACACA and PPARα, HepG2 cells were used to perform the in vitro experiments. Firstly, the safe dose ranges of these four polyphenol compounds were determined using cell viability assays on HepG2 cells. The highest concentrations of polyphenol compounds that resulted cell viability > 95% were determined as the best effective concentration. This was found to be 3.75, 2.375 and 3.5625 µg/mL for KAE, QUE, and HYE, respectively (Fig. 5A). These concentrations were then used to study their effects on the mRNA expression levels of MASLD-related genes in HepG2 cells.

### PE-CSLT exhibits regulatory effects on mRNA expression levels in HepG2 cells

As illustrated in Fig. 5B, the PEC group demonstrated a significant increase in the relative mRNA expression levels of AMPK compared to the CON group (*p* < 0.05). When compared to the SIM positive control group, the PEC group also showed a notable elevation in the relative mRNA expression of SREBP1 (*p* < 0.05). Both the PEC and HYP groups exhibited significantly higher mRNA expression levels of ACACA compared to the CON group (*p* < 0.05). However, in contrast to the hepatocyte steatosis model (MOD) group, the QUE group displayed a significant reduction in ACACA mRNA expression (*p* < 0.01), while the other three drug groups remained unchanged (*p* > 0.05). Compared to the SIM positive control group, the KAE and QUE groups had significantly decreased ACACA mRNA expression levels (*p* < 0.01 and *p* < 0.001, respectively). Furthermore, PPARα mRNA expression levels were significantly elevated in the PEC group compared to both the CON and MOD groups (*p* < 0.01), whereas, in comparison to the SIM group, the KAE group showed a significant reduction in PPARα mRNA expression (*p* < 0.05).

### Pharmacodynamics of PE-CSLT in HFD rat livers

#### PE-CSLT decreases body weight growth in MASLD rats

After 12 weeks of treatment, the body weights of the four groups CON, MOD, PEC and the PEM were (461.4 ± 20.56)g, (537.93 ± 14.36)g, (474.93 ± 39.12)g, (515.5 ± 41.6)g, respectively. Administration of PE-CSLT resulted in a decrease in the weights of PEM when compared to the rats in MOD (*p* < 0.05). Conversely, no significant differences in weights were observed between CON and PEC (*p* > 0.05). This indicated that PE-CSLT was effective in reducing body weight growth in MASLD rats that induced with HFD (Figs. 4B and 5A).

#### PE-CSLT ameliorates serum lipid lever in MASLD rats

**Fig. 6:**
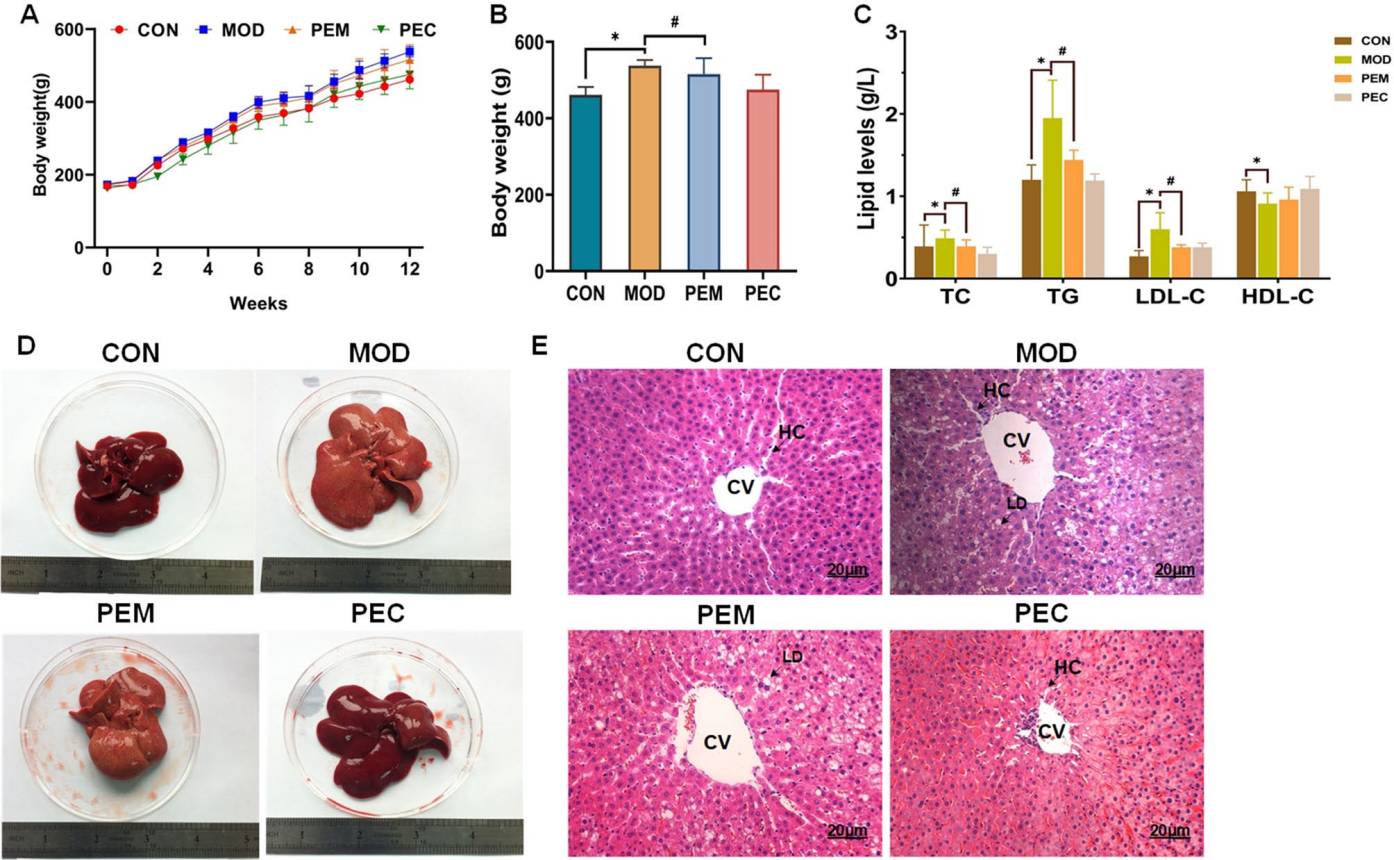
The hypolipidemic effects of the polyphenols extracted from CSLT on the livers of MASLD rats. (**A**) The effects of polyphenols extracted from CSLT on the body weights of MASLD rats. (**B**) The effects of polyphenols extracted from CSLT on the liver weight/body weight index of MASLD rats. (**C**) The effects of polyphenols extracted from CSLT on the lipid levels in sera of MASLD rats. (**D**) Morphological observations of the livers of rats in each group. (**E**) Light microscope observations of HE staining on liver tissue sections of MASLD rats (HE, ×200). The data are expressed as means ± SD (*n* = 3). *p < 0.05, when compared with CON group; #*p* < 0.05 when compared with MOD group;. CON: control, MOD: model, PEM: PE-CSLT control group, PEC: PE-CSLT model group, CV: Central Vein, HC: hepatic sinusoid, LD: Lipid droplet

Compared with the rats in the CON, the liver index of the MOD animals significantly increased (*p* < 0.05), and this improved after administration of PE-CSLT (*P* < 0.05; Fig. 6C). Compared with the CON, the serum levels of TC, TG and LDL-C in MOD were significantly increased (*p* < 0.05), and HDL-C was significantly reduced (*p* < 0.05), indicating that the MASLD model was successful (Fig. 6C). Compared with MOD, the serum levels of TC, TG and LDL-C decreased (*p* < 0.05) in PEM (Fig. 5C). There was some increase in the serum lever of HDL-C in PEM compared with MOD but not significant. The above results showed that PE-CSLT could effectively ameliorated the serum levels of TC, TG, and LDL-C in MASLD rats.

#### PE-CSLT relives liver lipid deposition in MASLD rats

It might be observed by HE staining that when comparing the MOD and PEM, the rats in MOD had fatty lesions in their livers, which there were increased lipid droplets in the cytoplasm of MOD (Fig. 6D and E). Compared with MOD, the liver fatty lesions in PEM were reduced, showing that PE-CSLT exhibited a partial efficacy in relieving lipid deposition in the livers of MASLD rats (Fig. 6E).

### Validation of mRNA expression levels of genes by HTGS

**Fig. 7:**
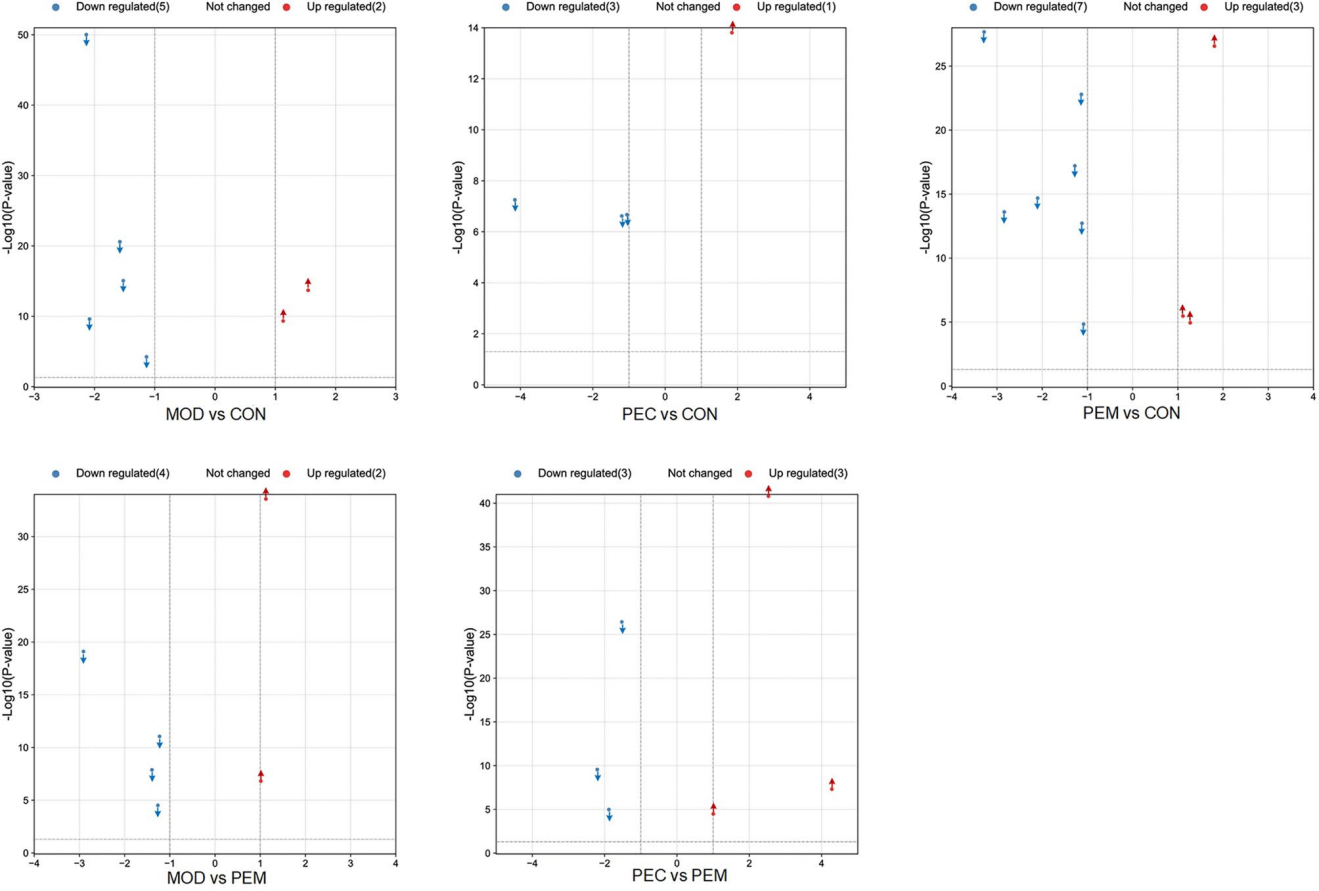
The effects of polyphenols extracted from CSLT on the differential gene expression of rat livers as evaluated by HTGS. The blue dots and arrows represent the down-regulated differential genes and the red dots and arrows represent the up-regulated differential genes. CON: control, MOD: model, PEC: PE-CSLT control group, PEM: PE-CSLT model group

The HTGS results from the four groups of rat livers revealed twelve differentially expressed genes (DEGs) linked to lipid metabolism (Fig. 7). In comparison to the CON group, the MOD group exhibited seven DEGs pertaining to lipid metabolism, with five genes being down-regulated and two being up-regulated. Upon administration of PE-CSLT to the CON group, ten DEGs related to lipid metabolism were identified. Specifically, seven genes were down-regulated, while three were up-regulated. When comparing the CON group before and after PE-CSLT administration, four DEGs emerged, with three genes down-regulated and one gene up-regulated. In contrast, the MOD group treated with PE-CSLT showed six DEGs, four down-regulated and two up-regulated. Furthermore, a comparison between the PE-CSLT-treated CON and MOD groups revealed six DEGs, with three genes down-regulated and three genes up-regulated. These specifically identified DEGs are intimately associated with MASLD, liver cirrhosis, diabetes mellitus, and obesity. They play roles in lipid metabolism, immunity, and cytokine signal transduction pathways. Notably, AMPK and PPARα demonstrated the highest|log2Fold change| value in the MOD group’s rat livers.

## Discussion

CSLT is a natural sweetener with high sweetness, low calorie content and is safe to use [14]. Polyphenols are one of the major active components of CSLT [15]. It has been reported that the PE-CSLT have pharmacological effects in being able to protect the liver as well as reduce the levels of fat and sugar in the blood, and it has the potential for treating fatty liver, obesity, diabetes and cardiovascular diseases [16]. In our comprehensive study, we delved into the therapeutic potential of polyphenols extracted from CSLT as novel therapeutic agents for MASLD. Our findings underscore the promising role of natural products in modern medicinal practices and provide valuable insights into their underlying molecular mechanisms.

### Therapeutic potential and molecular mechanisms of PE-CSLT in MASLD

The liver is a central organ that plays an important role in lipid metabolism [17]. MASLD is characterized by lipid metabolism disorders and excessive hepatic lipid accumulation [18]. This study systematically elucidated the therapeutic effects and molecular mechanisms of polyphenol extracts from CSLT in treating MASLD using integrated multi-omics approaches. Our findings demonstrated that PE-CSLT significantly upregulated the expression of AMPK, PPARα, and other energy metabolism-related genes while downregulating SREBP1 and ACACA, key regulators of lipogenesis. This dual regulatory effect establishes a therapeutic network combining “promotion of fatty acid oxidation” and “inhibition of lipid synthesis,” consistent with previous reports on the metabolic benefits of stevia components [19, 20].

### Core therapeutic targets and signaling pathways

Network pharmacology analysis identified the AMPK/SREBP1/PPARα axis as the central regulatory network underlying PE-CSLT’s therapeutic effects. As a master energy sensor, AMPK activation not only promotes mitochondrial fatty acid oxidation but also suppresses lipogenesis through SREBP1 inhibition [21, 22]. The coordinated regulation of PPARα, a nuclear receptor controlling fatty acid metabolism, and ACACA, the rate-limiting enzyme in fatty acid synthesis, demonstrates PE-CSLT’s multi-target intervention strategy [23, 24]. This mechanism aligns with contemporary therapeutic paradigms for metabolic disorders, emphasizing synergistic pathway modulation over single-target approaches.

### Synergistic effects of polyphenol components

The complex polyphenol composition of CSLT necessitates a thorough evaluation of individual compounds and their contributions to the overall therapeutic effects. Our study has identified eleven polyphenols with therapeutic potential, specifically quercetin, kaempferol, hyperoside, rutin, brevifolic acid, gallic acid, quercetin-3-O-L-arabinoside, ellagic acid, caffeic acid, and isoquercitrin, as active components of CSLT closely linked to MASLD targets. Notably, quercetin, kaempferol, and hyperoside demonstrated high connectivity in our network analysis, suggesting their potential as lead therapeutic candidates. This composition-activity relationship reflects the TCM principle of “monarch-minister-assistant-guide” herb combination, where multiple components act synergistically through distinct molecular targets.

### Safety evaluation and dose optimization

While CSLT has a long history of safe use as a food additive, this study established cell viability thresholds (CC_50_ = 53.50 µg/mL) and defined a safe therapeutic window (6.69–26.75 µg/mL) using MTT assays. Future studies should implement dose-response experiments (1.8–7.2 g/kg·d) to establish animal pharmacokinetic/pharmacodynamic relationships and optimize the therapeutic index. These doses align with preclinical dosing regimens reported in plant polyphenol research (100–500 mg/kg in rat/mice HFD models) [25–30].

### Methodological limitations and improvement strategies

While aligned with herbal research paradigms, the absence of FDA-approved positive controls (e.g., simvastatin) limits comparative efficacy assessment. Though mRNA-protein correlations (e.g., ATG7 *r* = 0.82 in human NASH) partially validate translational relevance, direct proteomic confirmation remains pending [31, 32]. Additionally, omission of molecular dynamics simulations precludes atomic-level insights into ligand-receptor dynamics. Future iterations will address these gaps through interdisciplinary collaborations incorporating computational biology and standard-of-care comparators, while implementing targeted proteomics to resolve post-transcriptional regulatory mechanisms. Such enhancements will bolster mechanistic rigor while maintaining relevance to botanical drug development frameworks.

### Translational research directions

This study lays the groundwork for three translational research avenues: (1) Phase I clinical trials focusing on MRI-PDFF-measured hepatic fat content and FIB-4 fibrosis scores; (2) CRISPR/Cas9-mediated gene knockout studies to elucidate SREBP1 mutation effects on lipid metabolism; and (3) fingerprinting of PE-CLST composition using AI-assisted spectral analysis for quality control and formulation optimization. These efforts will bridge the preclinical-clinical gap and establish PE-CSLT as a viable therapeutic option for MASLD.

## Conclusions

This study systematically evaluated the pharmacological mechanisms of PE-CSLT in MASLD using integrated pharmacology approaches, identifying 11 bioactive polyphenols and 12 lipid-regulating targets. Preclinical models demonstrated that PE-CSLT modulates AMPK/SREBP1/ACACA/PPARa signaling to attenuate hepatic steatosis and dyslipidemia, providing mechanistic support for its therapeutic potential. However, these findings represent early-stage preclinical investigations requiring validation in human-relevant systems before clinical extrapolation. Limitations include species-specific metabolic differences, the need for in-deepth experimental confirmation of computational predictions, and absence of long-term safety data. Future work should focus on human organoid models, structural mechanism dissection, and controlled clinical trials to rigorously assess translational value in MASLD management.

## Electronic supplementary material

Below is the link to the electronic supplementary material.

Supplementary Material 1

## Data Availability

No datasets were generated or analysed during the current study.

## Abbreviations

ACACA: Acetyl-CoA carboxylases alpha
ALT: Alanine transaminase
AMPK: 5’-AMP-activated protein kinase
AST: Aspartate transaminase
CSLT: Chinese sweet leaf tea
DEGs: Differentially expressed genes
DMEM: Dulbecco’s modified Eagle’s medium
DMSO: Dimethyl sulfoxide
FDRs: False Discovery Rates
HMGCR: Hydroxymethylglutaryl coenzyme A reductase
HTGS: High-throughput gene sequencing
HYP: Hyperoside
KAE: Kaempferol
L-PGDS: Lipocalin-type prostaglandin D synthetase
MASLD: Metabolic dysfunction associated steatotic liver disease
MTT: Thiazolyl blue tetrazolium bromide
NAFLD: Non-alcoholic fatty liver disease
OD: Optical density
PBS: Phosphate-buffered saline
PE: Polyphenols extract
PEC: PE-CSLT control group
PEM: PE-CSLT model group
PE-CSLT: Polyphenols extracted from Chinese sweet leaf tea
PPAR: Peroxisome proliferator-activated receptor
PPI: Protein-protein interaction
QUE: Quercetin
SREBF1: Sterol regulatory element binding transcription factor 1
SV: Simvastatin
TC: Total cholesterol
TG: Total triglyceride
