# Supplementary material for: The use of polyphenols extracted from Chinese sweet leaf tea (Rubus suavissimus S. Lee.) as novel drugs for the treatment of metabolic dysfunction associated steatotic liver disease
Source: Hereditas. 2025 Jul 24;162:141. doi: 10.1186/s41065-025-00504-6 (PMC12291371; doi:10.1186/s41065-025-00504-6)
Supplement: Supplementary file 1 — Supplementary Material 1 [file 41065_2025_504_MOESM1_ESM.docx]

**Supplementary Information**

**Table S1.** The primer sequences used for PCR in this study

| **Human Gene** | **Uniprot Entry** | **Protein Names** | **Primer Sequence** |
| --- | --- | --- | --- |
| AMPK/PRKAA2 | [P54646](https://www.uniprot.org/uniprot/P54646) | **5'-AMP-activated protein kinase catalytic subunit alpha-2** | F 5'-CCACATCTCCTCCAGGTCATCC-3’ |
|  |  |  | R 5’-GCTGAGCACCATCACTCCATCC-3’ |
| SREBAF1/SREBP1 | [P36956](https://www.uniprot.org/uniprot/P36956) | **Sterol regulatory element-binding protein 1** | F 5’-CACTGGTCGTAGATGCGGAGAA-3’ |
|  |  |  | R 5’-TCATTGATGGAGGAGCGGTAGC-3’ |
| ACACA | [Q13085](https://www.uniprot.org/uniprot/Q13085) | Acetyl-CoA carboxylase 1 | F 5’-CTGGCTGGCTGGACAGACTGAT-3’ |
|  |  |  | R 5’-ACGCTATTCCGCAGGCTCACA-3’ |
| PPARA | [Q07869](https://www.uniprot.org/uniprot/Q07869) | **Peroxisome Proliferator-activated receptor alpha, PPAR-alpha** | F 5’-AGCAAGGAAGGGTTGTGGCAAA-3’ |
|  |  |  | R 5’-ATGGACTCGGAAGCAGGAAGGT-3’ |
| GAPDH | [P04406](https://www.uniprot.org/uniprot/P04406) | GAPDH | F 5’-CCTGGATACCGCAGCTAGGA-3’ |
|  |  |  | R 5’-GCGGCGCAATACGAATGCCCC-3’ |
